# University of Maryland Dental Department

**Published:** 1894-04

**Authors:** 


UNIVERSITY OF MARYLAND DENTAL DEPARTMENT.—The Annual Commencement exercises of 1894 were held at the Lyceum Theatre, March 30th, 1894, at 3 P. M.

The Mandamus from the Board of Regents was read by the Dean, Prof. Ferdinand J. S. Gorgas, M. D., D. D. S. The address to the graduates was delivered by Rev. Dr. L. T. Townsend, of Mount Vernon M. E. Church. The Class Oration was delivered by C. Chandler George, D. D. S., of Illinois.

The Degree of "Doctor of Dental Surgery" was conferred upon the following gentlemen by Prof. R. Dorsey Coale, Ph. D., owing to the illness of the Hon. Severn Teackle Wallis, LL. D., Provost of the University (Mr. Wallis died April 11th, 1894):

Frank E. Blakeslee, N. Y.; William M. H. Burfeind, Cal.; James C. Chisholm, Ala.; Henry Clay Daniel, N. C.; Harry M. Dommett, N. Y.; Charles Augustus Davis, Wis.; Stanton D. Diffenderfer, Penn.; Thomas Dotterer, S. C.; Baylis D. Earle, S. C.; Milton S. Fleming, La.; H. Eugene Gee, S. C.; C. Chandler George, Ill.; Edward H. Goldberg, Md.; William L. Hartnett, Conn.; John E. M. Heffelbower, Texas; Phineas E. Horton, N. C.; Ambrose H. Huffman, W. Va.; William P. Lacy, Va.; William S. Lindsay, S. C.; Harter W. March, Ohio; Charles A. Matthews, Ind.; James I. Montgomery, S. C.; Redorick Morgan, N. C.; Leander B. Milbourne, Md.; James A. Proctor, Va.; William H. Renoe, Missouri; Clarence P. Reynolds, N. Y., C. Charles Stanley, S. C.; Norman R. Smithers, Md.; Fred. B. Smith, Penn.; Richard Channing Walden, Va.; John Eldridge Walton, Ga.; James Watson, Ill.; J. Brenton Wise, S. C.

The University Prize Gold Medal, for the highest number of votes at the final examinations, was awarded to Richard Channing

Walden, of Virginia. Honorable Mention: Harter W. March, of Ohio.

The S. S. White Dental Engine Prize was awarded to Wm. M. H. Burfeind, of Cal.

The Francis Arnold Prize, Set of Forceps, was awarded to Wm. M. H. Burfeind, of Cal.

The Snowden & Cowman Prize, Forceps of Chapin A. Harris patterns, was awarded to Wm. P. Lacy, of S. C.

The S. S. White Set of Varney's Pluggers was awarded to Wm. M. H. Burfeind, of Cal.

The Jas. Hart Gold Medal was awarded to Wm. M. H. Burfeind, of Cal.

Prof. Harris Prize was awarded to Jas. C. Chisholm, of Ala.

The Prof. Gorgas Prize was awarded to Wm. H. Renoe, of Mo.

The Dr. Jno. C. Uhler Prize was awarded to Wilton S. Fleming, of La.

The Dr. Isaac H. Davis Prize was awarded to Wm. M. H. Burfeind, of Cal.

The Dr. J. H. Marchant Prize was awarded to Norman R. Smithers, of Md.

The Jas. C. Chisholm Junior Prize was awarded to Delmar Smithers, of Md.

The Senior Class Prize was awarded to Jas. Brenton Wise, of S. C.

The Freshman Class Prize was awarded to Wm. A. Bisnare, of Canada.

The regular Winter Session of 1893-94 will close May 1st, 1894.

The Summer Session of 1894 will begin May 1st, 1894.

